# Obliteration of the Lacrymal Lac

**Published:** 1853-10

**Authors:** 


*Obliteration of the Lacrymal Lac.*

M. Magne, proposes, in cases of fistula lacrymalis, to obliterate the sac in the following manner: He reports several successful cases.

"With a double edged knife I open the sac, and enlarge the opening, if there is a fistulous orifice; I enlarge or separate the lips of the wound with an instrument which I call *speculum*, or dilator of the sac, I dissect and clear out the interior of the cavity; I then introduce the porte-caustique, and cauterise particularly the orifices of the Lacrymal ducts. The speculum of the sac answers the double purpose of dilating the lips of the wound, and of preserving the caustic from parts which should not be touched. A simple dressing, daily renewed, is all that is necessary. The operation is easily performed, and can be done in two minutes, requiring no assistance.

The epiphora, which follows for some time, ceases either by the evaporation of the tears from the surface of the eye, or from some modification in the gland, the result of the loss of a part of the lacrymal apparatus.—*Compte Rendu.*

---

The Queen of England has conferred the title of Baron upon Dr. Holland, one of her physicians-extraordinary.

We understand that Dr. G. A. Wilson, has received the appointment of Professor of Physiology and Medical jurisprudence in Hampden Sidney Medical College. Dr. Wilson is a Virginian; his views on medical education are liberal, and his abilities are highly esteemed by those who know him intimately. His appointment will doubtless contribute to the prosperity of the institution.

Dr. Fred. Robert Manson, physician-accoucheur to the Royal Pimlico Dispensary, was recently called upon to deliver a poor woman suffering from typhus fever. Dr. Manson had, at the time, a slight wound of the hand; through this wound the poisonous element was absorbed, erysipelas supervened, and death occurred nine days after the accident. The innocent cause of this distressing calamity has herself since died. Mr. Manson, whose untimely end we thus record, was a graduate in honors of the University of London, and a member of the College of Physicians.—*Virginia Med. and Surg. Journal.*

